# Impact of supplementation on deleterious mutation distribution in an exploited salmonid

**DOI:** 10.1111/eva.12660

**Published:** 2018-07-01

**Authors:** Anne‐Laure Ferchaud, Martin Laporte, Charles Perrier, Louis Bernatchez

**Affiliations:** ^1^ Institut de Biologie Intégrative et des Systèmes (IBIS) Université Laval Québec QC Canada; ^2^ Centre d’Écologie Fonctionnelle et Évolutive Unité Mixte de Recherche CNRS 5175 Montpellier Cedex 5 France

**Keywords:** conservation management, deleterious mutations, genetic diversity, Lake Trout (*Salvelinus namaycush*), stocking

## Abstract

Deleterious mutations have important implications for the evolutionary trajectories of populations. While several studies recently investigated the dynamics of deleterious mutations in wild populations, no study has yet explored the fate of deleterious mutations in a context of populations managed by supplementation. Here, based on a dataset of nine wild and 15 supplemented Lake Trout populations genotyped at 4,982 single nucleotide polymorphisms (SNP)s by means of genotype by sequencing (GBS), we explored the effect of supplementation on the frequency of putatively deleterious variants. Three main findings are consequential for the management of fish populations. First, an increase in neutral genetic diversity in stocked populations compared with unstocked ones was observed. Second, putatively deleterious mutations were more likely to be found in unstocked than in stocked populations, suggesting a lower efficiency to purge deleterious mutations in unstocked lakes. Third, a population currently used as a major source for supplementation is characterized by several fixed putatively deleterious alleles. Therefore, other source populations with lower abundance of putatively deleterious mutations should be favored as sources of supplementation. We discuss management implications of our results, especially pertaining to the joint identification of neutral and deleterious mutations that could help refining the choice of source and sink populations for supplementation in order to maximize their evolutionary potential and to limit their mutation load.

## 1 INTRODUCTION

New mutations arise in populations every generation. Beneficial mutations are generally viewed as the main driver of evolution through adaptation, but most species harbor many deleterious mutations that have not been eliminated by selection (Agrawal & Whitlock, 2012). In fact, most mutations affecting fitness are deleterious (Keightley & Lynch, 2003) and, excluding those with severe/lethal effects, mildly deleterious mutations are expected to accumulate and ultimately impact wild populations (mutation load; Kimura, Maruyama, & Crow, 1963). Therefore, understanding the mechanisms underlying the accumulation of deleterious mutations is of great interest and has led to studies testing the effects of mating systems, demography, domestication, recombination rate, and other genomic features on deleterious mutation trajectories (Bosshard et al., 2017; Charlesworth, 2012; Chen, Glémin, & Lascoux, 2017; Laenen et al., 2018; Peischl et al., 2017; Renaut & Rieseberg, 2015; Zhang, Zhou, Bawa, Suren, & Holliday, 2016; Zhou, Massonnet, Sanjak, Cantu, & Gaut, 2017). Systems in which genetic drift prevails over natural selection (e.g.*,* small population size) are of major concern for conservation and management programs because they may allow random deleterious variants to increase in frequency (Lande, 1994; Lynch, Conery, & Burger, 1995; Wright, 1931). Moreover, inbreeding can unmask recessive deleterious mutations, affecting fitness of individuals (Charlesworth & Willis, 2009). In wild populations, the presence of deleterious mutation has been recently found in small populations and used to evaluate the mutation load and the potential for accumulation of deleterious mutations (Benazzo et al., 2017; Perrier, Ferchaud, Sirois, Thibault, & Bernatchez, 2017). Predicting the functional effect of substitution is now possible and thus may be an important criterion to consider when implementing management actions that are currently not considered in conservation or management programs.

Lake Trout is an important recreational fish widely distributed in cold freshwater lakes of North America (Scott & Crossman, 1998). Pronounced genetic differentiation has been documented among natural/unstocked Lake Trout populations (Halbisen & Wilson, 2009; McCracken, Perry, Keefe, & Ruzzante, 2013; Northrup, Connor, & Taylor, 2010; Perrier et al., 2017; Piller, Wilson, Lee, & Lyons, 2005; Valiquette, Perrier, Thibault, & Bernatchez, 2014). Intrapopulation genetic diversity is typically low in this species (Bernatchez, Laporte, Perrier, Sirois, & Bernatchez, 2016; Valiquette et al., 2014) and positively correlated with lake size (Perrier et al., 2017). Together, they indicate the major role of genetic drift for determining the genetic diversity in lakes after their isolation following the last glacial retreat (Wilson & Mandrak, 2003). In a study dealing specifically on wild (unstocked populations), Perrier et al. (2017) detected 124 putative deleterious variants across all lacustrine populations they surveyed. The frequencies of such deleterious mutations, relative to the entire polymorphism within populations, were positively correlated with inbreeding, suggesting that the efficacy of purifying selection was negatively correlated with local level of inbreeding. Furthermore, approximately 46% of the Lake Trout populations in the southern part of province of Québec (Canada) have been stocked (mostly from wild populations), and the maintenance of several of these populations is considered dependent on stocking relative to fishing pressure (communication with the wildlife Québec agency, *Ministère de la Forêt, de la Faune et des Parcs*—MFFP). Based on 19 microsatellites markers, an increase in genetic diversity and a twofold decrease in genetic differentiation among stocked compared to unstocked populations were reported across Lake Trout populations (Valiquette et al., 2014). Furthermore, levels of admixture in stocked populations were correlated with stocking intensity, resulting in genetic homogenization of the heavily stocked populations (Valiquette et al., 2014). The potential effect of stocking on the accumulation of deleterious mutations has not been tested yet in Lake Trout nor in any other exploited fish species to our knowledge. The extensive analyses of the population genetic impacts of stocking and of the accumulation of deleterious mutations in wild Lake Trout populations have paved the way for asking the timely question of how stocking affects the accumulation of deleterious mutations, which is of broad relevance for stocking management in general.

The main objective of this study was to assess the effect of stocking on the accumulation of putative deleterious variants. Specifically, this study will answer this question: Do the putative deleterious variants accumulate differentially in unstocked and stocked populations? We achieved this objective using a total of 578 fish from 24 populations in Québec, Canada, that we genotyped using a large single nucleotide polymorphism (SNP) panel designed by a genotyping by sequencing approach. On the basis of the results obtained, we discuss how stocking practices could be adjusted toward improving their usefulness by maintaining the evolutionary potential of populations, and how the use of SNPs may help improving stocking practices by increasing power to decipher population structure and through the identification of deleterious mutations.

## 2 MATERIALS AND METHODS

### 2.1 Sampling, study system and molecular analyses

**Figure 1:**
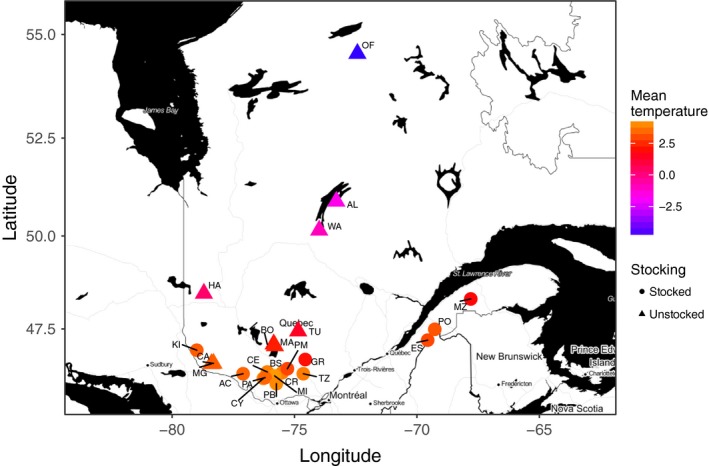
Geographic location of the 24 sampled Lake Trout populations in Québec (Canada). Gradient color corresponds to the average daily air temperature, and the symbols indicate whether the population has been stocked or not

Samples from 24 lakes from Québec (Canada) were selected from the study of Valiquette et al. (2014) (Figure 1, Table 1) to maximize the (a) representation of genetic groups previously identified (Valiquette et al., 2014), (b) variation in levels of stocking, and (c) quality of samples for performing GBS. Stocking history information was extracted from the MFFP database. Among selected lakes, nine have never been stocked while 15 have been stocked at least once (Table 1). It is worthwhile to mention that Lake Trout lakes are generally not supplemented from hatchery, but from wild fish from another lakes (Supporting Information Table S1). The median number of individuals per lake was 23, ranging from 10 to 41 (Table 1).

**Table 1:** Population code and corresponding lake size (in hectares), sample size (*N*), administrative region, latitude, longitude, altitude (in meters), and stocking state (see Supporting Information Table S1 for more details about stocking and air temperature)

Lake_code	Lake size (hr)	*N*	Region	Latitude	Longitude	Altitude	Stocking
CA	746	17	Abitibi‐Temiscamingue	46.54	−78.31	329	Unstocked
HA	39	36	Abitibi‐Temiscamingue	48.47	−78.7	363	Unstocked
MG	622	19	Abitibi‐Temiscamingue	46.53	−78.39	253	Unstocked
KI	30,044	17	Abitibi‐Temiscamingue	46.91	−78.99	269	Stocked
PA	109	30	Outaouais	46.14	−76.2	252	Stocked
CE	793	22	Outaouais	46.3	−76.11	181	Stocked
AC	673	28	Outaouais	46.25	−77.1	283	Stocked
CY	725	41	Outaouais	46.12	−76.27	194	Stocked
BS	1,437	30	Outaouais	46.22	−76.05	164	Stocked
MI	4,973	34	Outaouais	46.19	−75.81	159	Stocked
BO	531	17	Laurentides	47.08	−75.85	299	Unstocked
MA	919	18	Laurentides	47.03	−75.8	329	Unstocked
TU	352	34	Laurentides	47.44	−74.85	436	Unstocked
GR	137	19	Laurentides	46.65	−74.56	485	Stocked
PM	357	26	Laurentides	46.39	−75.29	317	Stocked
TZ	958	24	Laurentides	46.25	−74.64	226	Stocked
CR	1,267	15	Laurentides	46.28	−75.5	209	Stocked
PB	5,475	17	Laurentides	45.97	−75.74	198	Stocked
AL	40,663	15	Nord‐du‐Quebec	50.9	−73.29	390	Unstocked
OF	5,110	29	Nord‐du‐Quebec	54.55	−72.43	431	Unstocked
WA	8,184	25	Nord‐du‐Quebec	50.15	−73.99	463	Unstocked
ES	743	32	Bas‐St‐Laurent	47.19	−69.56	321	Stocked
PO	894	10	Bas‐St‐Laurent	47.49	−69.27	207	Stocked
MZ	1,864	23	Bas‐St‐Laurent	48.32	−67.8	271	Stocked

DNA was extracted from the same adipose fin clips for GBS genotyping using a modified version of salt extraction protocol (Aljanabi & Martinez, 1997). An RNAse A (Qiagen) treatment was applied following the manufacturer's recommendation. DNA quality was checked using agarose gel electrophoresis. DNA was quantified using a NanoDrop spectrophotometer (Thermo Scientific) and then using Quant‐iT Picogreen dsDNA Assay Kit (Invitrogen). GBS librairies were prepared following a modified version of the two‐enzyme GBS protocol, using *PstI* and *MspI* restriction enzymes (Poland, Brown, Sorrells, & Jannink, 2012), and single‐end 100‐bp‐length sequencing on Illumina HiSeq2000 platform was conducted for all samples as detailed in Bernatchez et al. (2016).

### 2.2 DNA sequencing, genotyping and genetic diversity

Cutadapt version 1.9.dev0 was used to remove adaptor sequences with a maximum error rate set to 0.2 and processed reads shorter than 80 bp were removed. Sequence quality was then inspected using FastQC 0.11.1 (Andrews, 2010). Libraries were demultiplexed and filtered for overall quality using the module *process_radtags* from Stacks version 1.30 (Catchen, Amores, Hohenlohe, Cresko, & Postlethwait, 2011; Catchen, Hohenlohe, Bassham, Amores, & Cresko, 2013). One mismatch in individual tags was allowed for quality and ambiguous barcodes. To discard Illumina adaptors at the end of the read, reads were trimmed to 80 bp (Pujolar et al., 2013). Sequence reads were aligned to the Rainbow Trout (*Oncorhynchus mykiss*) genome (Berthelot et al., 2014) using the GSNAP version 2015‐12‐31v.9 (Wu & Nacu, 2010). Stacks pipeline version 1.40 was then used to discover loci and call genotypes. *Pstacks* was used to extract the stacks aligned to the reference genome and to identify SNPs at each locus with a minimum depth coverage of four to report a stack. Ten individuals per population (individuals with the highest number of sequence reads) were used to build the catalog using *ctsacks*. Set of stacks were then searched against the catalog using *sstacks* performing gapped alignment and using the –g option. The *populations* module was used to call genotypes for loci having a minimum genotype likelihood of −10. Subsequent filtering was applied to retain SNPs that were biallelic, genotyped in a least 50% of the individuals of a given population, and had a global minor allele frequency (MAF) higher than 0.05. This filter was used to avoid the introduction of sequencing error in the dataset and to avoid restriction of MAF in local populations at the same time. Only a single SNP per locus was kept. All filtering details are presented in Supporting Information Table S2. The Stacks workflow used is available online (https://github.com/enormandeau/stacks_workflow), and conversion of VCF files was produced with stackr R package version 0.5.5 (Gosselin & Bernatchez, 2016), VCFTOOLS 0.1.14 (Danecek et al., 2011), and PGDSPIDER 2.0.7.2 (Lischer & Excoffier, 2012). At last, we retrieved microsatellite data for the individuals retained after filtering during the SNPs calling. SNP genomic diversity within populations was reported as population averages for (a) the number of polymorphic reads per individual, (b) nucleotide diversity among variant sites, (c) proportion of SNPs genotyped, and (d) proportion of polymorphic SNPs (allele frequency different from 0 or 1).

### 2.3 Removing markers under potential selection

Detection of putative outliers was performed using two approaches: (a) SNPs potentially under balancing and divergent selection were identified using BAYESCAN v.2.1 (Foll & Gaggiotti, 2008) and (b) SNPs under polygenic selection linked to temperature were detected using a random forest algorithm (Boulesteix, Janitza, Kruppa, & Konig, 2012; Chen & Ishwaran, 2012; Goldstein, Polley, & Briggs, 2011). This second step was produced because different seasonal temperature variables may impose different selective pressures on Lake Trout (Perrier et al., 2017). The putative outliers were subsequently removed from the SNP datasets, such that only putative neutrals remained (hereafter called “neutral markers”) for the subsequent analyses. Details of Bayescan and random forest are provided in Supporting Information Data S1.

### 2.4 Heterozygosity, neutral genetic differentiation, and structure

GENEPOP version 4.2 (Raymond & Rousset, 1995; Rousset, 2008) was used to estimate observed and expected heterozygosities (H_O_ and H_E_, respectively), and a *t* test was performed between H_E_ of unstocked versus stocked populations. The R package assigner v.0.3.9 (Gosselin, Anderson, & Bradbury, 2016) was used to estimate pairwise *F*_ST_ (Weir & Cockerham, 1984) across all populations on both the neutral loci and the putatively deleterious mutation datasets and tested for significance with 1,000 bootstraps. Estimations of neutral admixture proportions of SNPs were performed with a discriminant analysis of principal components (DAPC). Because PCA methods do not accept missing data, imputations by lake were performed using the random forest algorithm implemented in the function “*genomic_converter”* from the “stackr” R package version 0.5.5. Then, the function “*find.clusters”* from the “adegenet” R package version 2.0.1 (Jombart & Ahmed, 2011) was used to assess the optimal number of groups with the Bayesian information criterion (BIC) method, considering no *prior* on group individual populations and allowing a maximum of 30 clusters. At last, the posterior individual assignment probabilities to each group (Q matrix) were obtained running the “*dapc”* function using the optimal number of discriminant functions to retain the optimal α‐score obtained from each dataset (Jombart, Devillard, & Balloux, 2010).

### 2.5 Identifying deleterious mutations

Deleterious mutations were first estimated by performing a BLAST on all SNPs against the Rainbow Trout transcriptome using blastx. We used the Rainbow Trout transcriptome because it was the best transcriptome available among salmonids at the time we produced this work and for the sake of similarity with Perrier et al. (2017). All hits with a similarity higher than 25 amino acid of 26 possible (considering 80‐bp‐long read) and more than 95% similarity between the query read and the transcriptome sequence were retained. As described in Perrier et al. (2017), we then identified the nonsynonymous mutations across the significant hits. As the prediction of the functional effect of amino acid changes may depend on the direction (i.e., the predicted effect of Gly to Asp does not equal an Asp to Gly change), for each nonsynonymous substitution, we identified the specific amino acid substitution and reported the structural/physicochemical category to each amino acid according to the classification reported in Supporting Information Table S3.

**Figure 2:**
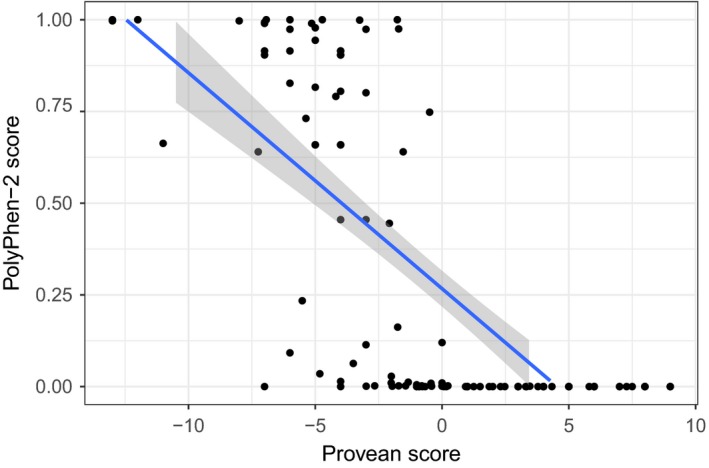
Relationship between Provean and PolyPhen‐2 scores. Only loci for which scores have been collected for both methods were considered for this comparison (130 of 214 nonsynonymous mutations)

We employed PROVEAN (Protein Variation Effect Analyzer; Choi, Sims, Murphy, Miller, & Chan, 2012) and PolyPhen‐2 (Adzhubei et al., 2010) to predict a potential damaging effect of a missense mutation. Using linear model in R, we tested the correlation between PolyPhen‐2 and Provean scores. The results inferred from the two methods were highly congruent (adj. *R* ^2^ = 0.52; *p* < 0.001; Figure 2 and Supporting Information Table S4), and we therefore show only PROVEAN scores for the subsequent analyses because unlike PolyPhen‐2, this method is not limited to human genes. Provean scores correlate with biological activity and may be used as an indicator for the degree of functional impact of a given protein variation. We investigated whether the values of these scores were significantly different across changes of amino acid categories by conducting two ANOVAs. In the first one, the direction of a category to another was kept whether in the second ANOVA, we clustered together two changes that implied the same categories with the goal of confirming the importance of the directionality of change on the potential deleterious state of a mutation.

At last, a default deleterious threshold value of −2.5 in Provean scores, as commonly employed by other studies (e.g.*,* Renaut & Rieseberg, 2015), was used to classify a nonsynonymous mutation as potentially deleterious (<−2.5) or neutral (>−2.5). We thus applied Provean scores to test whether local deleterious allele frequencies (considered as the minor allele frequency of SNPs harboring a putative deleterious mutation) differ among populations using a multivariate analysis of variance (MANOVA). Next, the proportion of deleterious mutations (defined as the number of SNPs showing a deleterious mutation in a given population over the number of SNPs harboring a deleterious mutation across all populations) and the ratio of the proportion of deleterious mutations over the proportion of polymorphic SNPs were estimated and compared among populations. The latter ratio is expected to be lower in populations that purged deleterious mutations faster (see Perrier et al., 2017) and was compared between stocked and unstocked populations using a *t* test. Because deleterious alleles are negatively selected, it is expected that their frequencies should be lower than nondeleterious ones (Fay, Wyckoff, & Wu, 2001). Thus, *t* tests were also used to verify whether average “deleterious allele frequency” is lower than the ones of “nonsynonymous, but nondeleterious SNPs” and to the ones of “synonymous SNPs” within each stocked and unstocked groups.

### 2.6 Effect of stocking on neutral genetic variation and accumulation of deleterious mutations

The effects of stocking and spatial autocorrelation on the genetic variation of both neutral genetic structure and deleterious alleles were tested using a distance‐based redundancy analysis (db‐RDA; Legendre & Legendre, 1998). First, we estimated a pairwise *F*_ST_ distance matrix (neutral loci or loci with potential deleterious mutations) and produced principal coordinates analyses (PCoAs) on this pairwise *F*_ST_ distance matrix to obtain the matrix to be explained by the db‐RDA tests. For neutral SNPs, the Q matrix (i.e.*,* admixture proportions; DAPC averaged at population level) was also used to serve as proxies for genetic structure to be explained by a db‐RDA test. Only PCo‐axes explaining at least 5% of the variance were considered meaningful and kept. As a proxy for stocking intensity, a matrix of the total weight (in kg) of fish stocked from 13 sources (column) to the 24 studied lakes (row) was extracted from the MFFP database (Supporting Information Table S1). Because the density of stocked fish in a lake is a good indicator of stocking pressure (Létourneau et al., 2017), each row was divided by the surface of the stocked lake in hectare. Furthermore, a Hellinger transformation was produced to avoid the effect of absence of stocking on the similarity among objects (Legendre & Legendre, 1998). A PCA was produced on the transformed database and a broken‐stick distribution was thereafter used to choose the meaningful axes related to stocking pressure. To control for spatial autocorrelation, a distance‐based Moran's eigenvector map (db‐MEM) based on latitude, longitude, and altitude, all pretransformed in meter with the function “*geoXY”* of the R package “SoDA,” was produced. The PC‐axes related to stocking intensity and db‐MEMs related to spatial components constituted the two explanatories matrices. To identify the best model, the function “*ordistep”* was used to select the best explanatory variables among stocking and spatial matrices. The function “*rda”* was finally used to compute the db‐RDAs on the selected model (see Benestan et al., 2016; Laporte et al., 2016; Le Luyer et al., 2017; Marengo et al., 2017, for examples of similar methodology). In addition, to ensure that stocking variables explained a significant part of the variation, a partial db‐RDA analysis was produced to test for the effect of the stocking variables after controlling for the spatial autocorrelation (db‐MEMs). An analysis of variance (ANOVA; 1,000 permutations) was then performed to assess the global significance of the db‐RDAs and partial db‐RDAs, and the percentage of variance explained (PVE) was computed with the function “*RsquareAdj.”* When not mentioned, R functions were part of the “vegan” package.

### 2.7 Comparison SNPs/microsatellites

Single nucleotide polymorphism database produced in this study contained the exact same individuals that the one of Valiquette et al. (2014) based on 19 microsatellites. We thus took advantage of both of the large dataset to compare their respective capacity to detect an effect of stocking on neutral genetic variation. Because results were similar between both datasets (although SNPs showed an increase in power given the much higher number of loci screened), this section is only presented in Supporting Information Data S2 for sake of comparison.

## 3 RESULTS

### 3.1 DNA sequencing, genotyping, and genetic diversity

The total number of demultiplexed and cleaned reads was 1,143,048,513 with an average of 821,578 reads per individual. After DNA control quality and filtering out individuals with more than 30% of missing genotypes, 578 of 629 individuals were kept for subsequent analyses. The assembly with the Rainbow Trout reference genome resulted in a catalog containing 1,359,992 distinct SNPs. After filtering for quality and keeping only a single SNP per locus, 4,982 SNPs were retained (Supporting Information Table S5). The median value of SNPs genotyped per population was 4,335 (Table 2), and the median depth of coverage was 35. The median number of polymorphic SNPs per population was 3,178 (64%); the median value of Pi was 1.29E‐03, ranging from 9.56E‐04 (pop HA) to 1.49E‐03 (pop PM, Table 2).

**Table 2:** Descriptive genetic statistics for each sampling locality. Observed and expected heterozygosity for both all SNPs (H_O_ SNPs, H_E_ SNPs) and neutral SNPs (H_O_ neutral SNPs and H_E_ neutral SNPs), genotype ratio, nucleotide diversity (Pi), number of polymorphic SNPs, proportion of deleterious mutations; ratio of deleterious mutations over ratio of polymorphic SNP, and the average minor allele frequency of deleterious alleles and number of fixed deleterious alleles

Lake code	Genotype ratio	Pi	H_O_ SNPs	H_E_ SNPs	Polymorphic SNPs	H_o_ neutral SNPs	H_E_ neutral SNPs	Polymorphic neutral SNPs	Deleterious ratio	Ratio of deleterious mutations over ratio of polymorphic SNP	Average minor allele frequency of deleterious alleles	Number of fixed deleterious alleles
CA	0.88	0.00107	0.24	0.17	2454	0.16	0.13	2454	0.57	1.15	0.15	0
HA	0.87	0.000956	0.21	0.15	2596	0.13	0.11	2596	0.56	1.07	0.13	0
MG	0.9	0.00101	0.24	0.16	2425	0.15	0.12	2425	0.54	1.11	0.13	0
KI	0.84	0.00121	0.25	0.19	2936	0.18	0.17	2936	0.58	0.98	0.14	0
PA	0.86	0.00129	0.27	0.2	3412	0.20	0.18	3412	0.6	0.88	0.13	0
CE	0.86	0.00128	0.26	0.2	3145	0.19	0.18	3145	0.59	0.94	0.13	0
AC	0.88	0.0013	0.27	0.21	3449	0.20	0.18	3449	0.67	0.97	0.13	0
CY	0.9	0.00135	0.27	0.21	3534	0.21	0.19	3534	0.64	0.9	0.13	0
BS	0.89	0.00136	0.29	0.22	3543	0.22	0.19	3543	0.67	0.95	0.14	0
MI	0.91	0.00131	0.29	0.21	2979	0.22	0.19	2979	0.58	0.96	0.14	0
BO	0.89	0.00111	0.25	0.17	2569	0.17	0.14	2569	0.58	1.12	0.14	0
MA	0.92	0.00125	0.28	0.2	2962	0.20	0.17	2962	0.56	0.94	0.14	0
TU	0.86	0.00113	0.26	0.17	2549	0.18	0.14	2549	0.69	1.35	0.24	3
GR	0.85	0.00137	0.28	0.22	3608	0.21	0.20	3608	0.67	0.93	0.14	0
PM	0.87	0.00149	0.31	0.24	3610	0.25	0.22	3610	0.72	0.99	0.23	3
TZ	0.87	0.00145	0.3	0.23	3440	0.24	0.21	3440	0.71	1.03	0.23	2
CR	0.87	0.00137	0.28	0.22	3520	0.21	0.20	3520	0.66	0.94	0.14	0
PB	0.9	0.00128	0.28	0.2	3087	0.21	0.18	3087	0.58	0.94	0.13	0
AL	0.9	0.00136	0.28	0.22	3326	0.22	0.20	3326	0.61	0.91	0.14	0
OF	0.87	0.00127	0.27	0.2	3046	0.20	0.18	3046	0.54	0.88	0.14	0
WA	0.85	0.00143	0.3	0.23	3462	0.24	0.21	3462	0.73	1.04	0.23	3
ES	0.87	0.00125	0.26	0.2	3415	0.19	0.17	3415	0.62	0.9	0.12	0
PO	0.89	0.00143	0.29	0.23	3610	0.22	0.21	3610	0.72	0.99	0.22	1
MZ	0.87	0.00129	0.29	0.2	3603	0.22	0.18	3603	0.64	0.88	0.13	0

### 3.2 Removing markers under potential selection

Among the 4,982 SNPs, BAYESCAN identified 437 SNPs (8.8%) that were potentially under divergent selection and 722 SNPs (14.4%) under balancing selection (Supporting Information Data S1; Figure S1.1). Analyses with the random forest algorithm selected 135 and 139 SNPs associated with temperature for population structure with *K* = 21 and *K* = 24, respectively (Supporting Information Data S1). In each case, model performance was not improved by reducing the number of selected loci according to the cross‐validation. Subsequently, 3,557 SNPs were defined as neutral SNPs (Supporting Information Figure S1).

### 3.3 Heterozygosity, neutral genetic differentiation, and structure

The median estimates of the observed and expected heterozygosity (H_O_ and H_E_) were, respectively, 0.20 and 0.18 for the neutral SNPs. The mean H_E_ was significantly higher among stocked populations (mean H_E SNPs stocked_ = 0.19) than among unstocked populations (mean H_E SNPs unstocked_ = 0.16, *p*_*t*.test_ = 0.016).

A pronounced pattern of population structure was observed among populations both Fst estimations and DAPC analyses. For neutral SNPs, pairwise *F*_ST_ ranged from 0.01 to 0.50 (mean = 0.22) and mean pairwise *F*_ST_ per population ranged from 0.15 (pop GR) to 0.36 (pop HA) (Supporting Information Data S2; Figure S2.1). The DAPC further confirmed the pronounced genetic structure observed among populations. The BIC values decreased as the number of K increased until *K* = 24 (Supporting Information Data S2; Figure S2.2). Thus, *K* = 24, which also corresponds to the total number of sampled populations, was used to estimate the posterior individual probabilities of assignment (Q matrix; Supporting Information Data S2; Figure S2.3).

### 3.4 Identifying deleterious mutations

**Figure 3:**
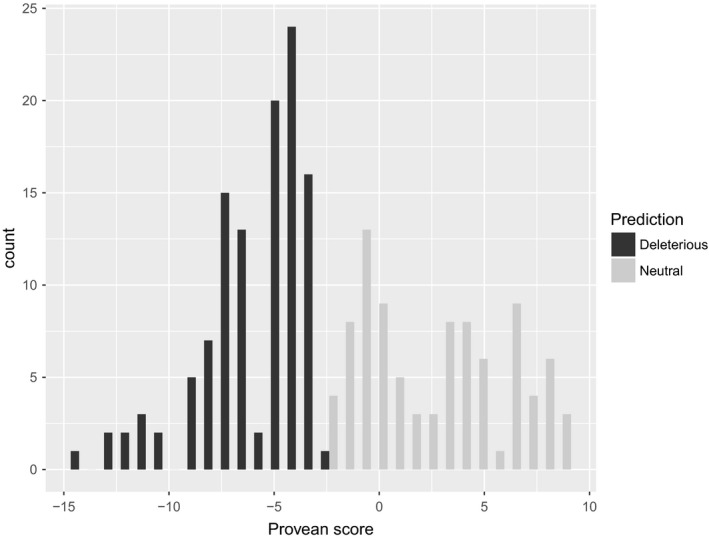
Distribution of PROVEAN scores among the 203 detected nonsynonymous mutations. The defaults threshold (−2.5) was used to detect 90 putatively neutral mutations and 113 putatively deleterious

**Figure 4:**
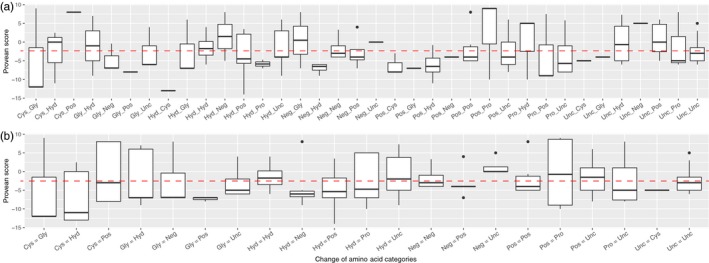
Boxplots of Provean scores according to the amino acid category considering (a) 36 different classes of substitutions (directionality of the mutation was considered) or (b) 21 different classes of substitutions (directionality of the mutation was not considered). The dashed red line indicates the Provean default threshold below which the substitution is considered potentially deleterious. Abbreviations correspond to what it is referenced in Supporting Information Table S3 (i.e.*,* Pos, amino acid with positive electrically charged side chains; Neg, amino acid with negative electrically charged side chains; Unc, amino acid with polar uncharged side chains; Hyd, amino acid with hydrophobic side chain; Cys, cystein; Sel, selenocysteine; Gly, glycine; Pro, proline)

Among all the 4,982 genotyped loci, 529 loci had significant BLAST results against the Rainbow Trout transcriptome (Berthelot et al., 2014) and were retained to assess synonymy. Synonymous substitutions were identified for 315 SNPs and nonsynonymous for 214 SNPs. Among the 214 nonsynonymous mutations, 90 were predicted to be neutral and 113 (52.8% of all nonsynonymous SNPs) were predicted to be putatively deleterious (Figure 3). The eight remaining mutations could not be attributed to one or the other category. Those nonsynonymous substitutions corresponded to 36 different classes of amino acid category changes or were restricted to 21 classes when pooling together classes implying twice the same two categories independent of the direction of the substitution. Considering the 36 classes, Provean scores were significantly different across classes (*df* = 35, *F* = 1.627, *p* = 0.022; Figure 4a), whereas when masking the direction of substitution (i.e.*,* considering 21 classes), the scores no longer differed significantly across classes (*df* = 20, *F* = 1.094, *p* = 0.359, Figure 4b), thus highlighting the importance of directionality in amino acid changes.

**Figure 5:**
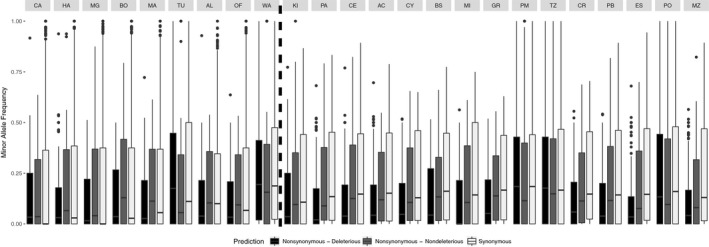
Boxplots of minor allele frequency of SNPs according three categories: nonsynonymous deleterious SNPs, nonsynonymous nondeleterious SNPs, and synonymous SNPs. Lakes represented to the left of the black dashed line are unstocked populations, whereas the 15 lakes represented to the right of the line correspond to stocked populations. Then, in each side of the line, lakes were placed according to their geographical proximity

MANOVA revealed a significant effect of the population factor on deleterious allele frequencies (*df* = 23, *F* = 4.81, *p* < 0.001), indicating that the average frequency of deleterious alleles was significantly different among populations. The proportion of deleterious mutations varied from 0.54 to 0.73 among populations (Table 2) with a median value of 0.62. The ratio of the proportion of deleterious mutations over the proportion of polymorphic SNPs per population varied from 0.88 (pop MZ) to 1.35 (pop TU). Moreover, the relative abundance of deleterious alleles was significantly lower in stocked than in unstocked populations (mean ratio_stocked_ = 0.94, mean ratio_unstocked_ = 1.06; *t* = −2.3801; *df* = 8.863; *p* = 0.040). Among stocked populations, the average local minor allele frequency for deleterious mutations (0.15) was significantly lower than “nonsynonymous but nondeleterious” SNPs (0.20; *df* = 2772.4; *t* = −5.6568; *p* < 0.001) as well as “synonymous” SNPs (0.23, *df* = 3297.5, *t* = −12.666, *p* < 0.001). Among unstocked populations, these differences were less pronounced but still significant (minor allele frequency: deleterious mutations = 0.16, nonsynonymous/nondeleterious = 0.19, synonymous = 0.20; *t* test results were respectively *df* = 1677.6; *t* = −3.04; *p* = 0.002; and *df* = 1104.2; *t* = −6.83; *p* < 0.001). The average proportion of minor allele for deleterious SNPs (i.e.*,* predicted deleterious alleles) with a frequency lower than 5% was 52% across lakes (ranging from 34% (lake TU) to 61% (lake PA). This proportion of minor allele with low frequency (<5%) was higher than the same proportions observed for both nonsynonymous/nondeleterious and synonymous polymorphisms (respectively 41% (from 34% (lake CR) to % (lake MG)) and 36% (from 30% (lake AL) to 42% (lake MG)); Figure 5). Together, these results suggest that deleterious mutations are more likely to be lost than putatively neutral polymorphisms (especially in stocked versus unstocked populations) but that there are still relatively common across all populations. At last, it is important to note that certain deleterious mutations were fixed in five populations, including an important source population used for stocking other lakes (TZ Lake, Table 2).

### 3.5 Effect of stocking on neutral genetic variation and accumulation of deleterious mutations

Based on the broken‐stick distribution, four stocking PC‐axes were considered meaningful and thus retained. Together, these explained 82.5% of the variance of our dataset for stocked populations. The loading factors revealed that the first stocking PC‐axis was associated with the source lakes 31‐Miles (*r* = 0.67) and Blue Sea (*r* = 0.70), the second stocking PC‐axis with the source lake Tremblant (TZ) (*r* = 0.81), and the third stocking PC‐axis with the source lake Mitis (MZ) (*r* = 0.65; Supporting Information Table S6). No stocking source was highly associated (correlation >0.60) with the fourth stocking PC‐axis (Supporting Information Table S6). In addition, 16 axes were obtained from the db‐MEM analysis (Supporting Information Table S7), which were all considered to test for the occurrence of spatial autocorrelation in all our RDA tests. For all variables retained of all RDA tests, maximum variance inflation factors were all under six (with majority under two), which is also under the suggested threshold of 10 (Hair, Black, Babin, Anderson, & Tatham, 1998).

For pairwise *F*_ST_ distance matrix, five PCo‐axes (totalizing 60% of explained variance) each explaining at least 5% of the variance were kept. Global model retained three spatial variables (Db‐MEM # 2,9 and 14) and one stocking variables (PC‐3, associated with source lake Mitis) and explained 24.5% of the five PCo‐axes (*p* < 0.001). A total of 15.7% of the variation was explained by the stocking variable alone after controlling for spatial variables (*p* < 0.01). For the Q matrix, 12 PC‐axes (totalizing 61% of explained variance) each explaining at least 5% of the variance were kept. Global model retained five spatial variables (Db‐MEM # 1, 2, 10, 12 and 14) and all four stocking variables and explained 26.5% of the 12 PCo‐axes (*p* < 0.001). A total of 16.9% of the variation was explained by the stocking variables after controlling for spatial variables (*p* < 0.001).

For deleterious mutations, two PCo‐axes (totalizing 63.3% of explained variance) each explaining at least 5% of the variance in pairwise *F*_ST_ distance matrix were kept. The global model retained one spatial variable (Db‐MEM #1) and one stocking variable (PC‐2, associated with source lake Tremblant), and marginally explained 13.2% of the five PCo‐axes (*p* < 0.1). A total of 8.8% of the variation was also marginally explained by the stocking variable alone after controlling for spatial variables (*p* < 0.1).

## 4 DISCUSSION

The main goal of this study was to assess the stocking impacts on the accumulation of putative deleterious mutations in an exploited fish, Lake Trout (*Salvelinus namaycush*). Our results highlighted an increase in neutral genetic diversity and a lower number of deleterious mutations in stocked populations compared with unstocked ones, as well as an effect on deleterious mutation variation related to a major source for supplementation characterized by several fixed putatively deleterious alleles (stocking PC‐2, Tremblant lake). Below we discuss the usefulness of GBS for monitoring putative deleterious alleles in source and stocked populations in order to mitigate potential harmful effects linked to stocking practice.

### 4.1 Effect of stocking on neutral genetic diversity and variation

An increase in genetic diversity (at both marker types, see Supporting Information Data S2 for a detailed discussion on this topic) was observed in stocked compared with unstocked lakes, as already reported by Valiquette et al. (2014). However, the percentage of variance of genetic differentiation (*F*_ST_) or admixture matrix explained by stocking had not been explored in previous studies to our knowledge. After controlling for spatial autocorrelation, we found a significant effect of stocking on both pairwise genetic differentiation (15.7% of variance explained) and the admixture matrix (16.9% of variance explained). It is interesting that only the stocking PC‐3 (source lake Mitis) showed an effect on pairwise genetic differentiation, whereas all stocking PC showed an effect on admixture matrix. This could be explained by a pronounced effect of genetic homogenization by stocking from Mitis Lake, which was previously reported by Valiquette et al. (2014). In the current study, the average pairwise *F*_ST_ for the three stocked populations using Mitis Lake as a stocking source (all from “*Bas‐St‐Laurent”* area) was 0.10 in comparison with 0.22 for all other populations. Such important genetic homogenization may explain why other stocking effects could not be detected by the multivariate analyses on pairwise Fst in comparison with Q matrix.

### 4.2 Detection and analyses of putative deleterious mutations

Our empirical GBS data on a nonmodel species highlights the fact that the prediction of the functional effect of amino acid change actually depends on the direction of amino acid substitution as suggested by Vasemägi et al. (2017). For several categories, one direction exhibits mainly “neutral” nonsynonymous mutations, whereas the other direction presents mainly putative deleterious mutations. A total of 113 putative deleterious SNPs was detected in our dataset corresponding to almost 53% of all nonsynonymous variations, which is in accordance with the 124 (60%) deleterious variants detected with a different dataset of natural Lake Trout populations (Perrier et al., 2017). For comparison, the percentage of nonsynonymous sites estimated to be deleterious in other species ranges from 3% in bacterial populations (Hughes, 2005) to 80% in the human genome (Fay et al., 2001). Such relatively abundant levels of deleterious mutations in Lake Trout populations could be attributed to their colonization history and to their small effective population sizes (Perrier et al., 2017), which are known to accumulate deleterious variants over time (Allendorf, Aitken, & Luikart, 2013; Balick, Do, Cassa, Reich, & Sunyaev, 2015; Benazzo et al., 2017; Frankham, Ballou, & Briscoe, 2010; Renaut & Rieseberg, 2015). Admittedly, the putative harmful mutations detected could also partly be linked to gene duplication and residual tetrasomic inheritance present in all salmonids due to their whole‐genome duplication (WGD) (Dehal & Boore, 2005; Jaillon, Aury, Brunet, Petit, & Stange‐Thomann, 2004; Macqueen & Johnston, 2014). Nevertheless, a recent study demonstrated that gene duplication can also impart fragility, not only robustness over mutations (Diss et al., 2017), suggesting that even duplicated regions remain strong candidates to harbor deleterious mutations. Furthermore, deleterious mutations should be kept at low frequency by natural selection (Kimura, 1983; Whitlock, Griswold, & Peters, 2003). The average proportion of minor alleles at potential deleterious SNPs with a frequency lower than 5% was more important than the proportion of nonsynonymous/nondeleterious and synonymous polymorphisms for both stocked and unstocked populations. These observations are consistent with the action of an effective purifying selection (Fay et al., 2001). Nevertheless, those rare predicted deleterious alleles frequencies varied among populations and some of them segregate at high frequency in a few populations, suggesting that the monitoring of markers harboring putative deleterious mutations has the potential to improve stocking practices, for instance for guiding the choice of sources and sinks for supplementation.

### 4.3 Effect of stocking on deleterious mutations

It is interesting that the average relative abundance of putative deleterious alleles over the entire polymorphism found in a population is higher in unstocked than stocked populations. In stocked populations, the addition of new individuals within a population from another lake could indeed both diminish the effect of drift as well as the subsequent fixation of putative deleterious, whereas in unstocked populations, genetic drift remains the main driver allowing putative deleterious variants to increase in frequency as observed in natural Lake Trout populations (Perrier et al., 2017) and in small populations of another species (Benazzo et al., 2017). Nevertheless, the potential of the several harmful impacts of stocking must be kept in mind, including genetic homogenization, loss of genetic integrity, and the weakening of local adaptation (Halbisen & Wilson, 2009; Lamaze, Garant, & Bernatchez, 2012; Marie, Bernatchez, & Garant, 2010; Valiquette et al., 2014). Thus, the use of stocking to potentially decrease deleterious mutations frequencies could generate other critical problems in natural populations. In addition, phenotypic evidence (e.g.*,* fluctuating asymmetry and developmental survival rate) supporting the deleterious state of those 113 candidates would be necessary to ensure that stocking could be beneficial to purge deleterious effect linked to low genetic diversity.

The redundancy analysis revealed that after controlling for spatial autocorrelation, a marginally significant effect of stocking (second stocking PC‐axis, associated with source lake Tremblant; PVE = 8.8%) was observed on the variation of deleterious alleles. This supports the idea that stocking has had an impact on putative deleterious mutations frequencies. Lake Tremblant showed the fourth highest ratio of potential deleterious SNPs across all populations and was the highest across source populations (71% of the 113 putative deleterious SNPs are present in this population). Moreover, this lake is also characterized by the second highest average frequencies of potential deleterious alleles across all populations and the highest among source populations (average minor alleles’ frequencies of 0.23). It was also the only source population with fixed deleterious mutations. Its ratio of potential deleterious mutations over ratio of polymorphic SNPs exceeded the value of 1, suggesting a limited potential to purge deleterious alleles. Altogether, this suggests that the marginal effect of Lake Tremblant population observed by the redundancy analysis could be detrimental for stocked populations. This result highlights the importance of estimating the variability of deleterious alleles in all source populations to avoid the anthropogenic spreading of deleterious mutations.

## 5 CONCLUSIONS AND MANAGEMENT RECOMMENDATION

On the basis of a comprehensive empirical dataset, we presented the first study answering the timely question of how stocking can affect the accumulation of putative deleterious mutations and consequently addressed recommendations for Lake Trout management. Our results suggest a slightly more effective purifying selection on deleterious variation in stocked than in unstocked populations. Nevertheless, stocking may cause harmful effects on wild populations, particularly when source populations are adapted to different environments (i.e., hatcheries or remote populations inhabiting a distinct environment). In natural Lake Trout populations, lake size was positively associated with genetic diversity (Perrier et al., 2017), which points to the importance of avoiding small size lakes as stocking sources. In this study, an effect of stocking associated with the source lake Tremblant was observed on deleterious mutations variability. Across our sampling of lakes, Tremblant is a medium size lake that showed relatively high frequency/fixed deleterious mutations. In addition, this lake appears to have difficulty purging its deleterious mutations, which overall suggests that fish supplementation from this source is not desirable and should be avoided. This result highlights the importance of assessing the genetic variability (including neutral and maladaptive alleles) before choosing a source for stocking and to avoid the spreading of deleterious mutations in the wild. Moreover, choosing a stocking source from the same area with similar environmental conditions should be favored with the goal to minimize potential impacts on recipient population's local adaptation. However, local adaptation is not always related to geography. For instance, ecotypic variation, such as piscivorous and planktivorous Lake Trout, could be intermingled across large geographic areas (Bernatchez et al., 2016). Therefore, phenotypic traits such as diet regime and growth rate could also represent other important cues to be considered before choosing a population as a source of stocking in Lake Trout.

## CONFLICT OF INTEREST

The authors declare no conflict of interests.

## AUTHORS CONTRIBUTION

L.B. and C.P. conceived the study. C.P. carried out laboratory work. A.‐L.F. did bioinformatic analyses. A.‐L.F. and M.L. carried out statistical analyses and wrote the manuscript. All co‐authors critically revised and contributed to editing the manuscript and approved the final version to be published.

## DATA ARCHIVING STATEMENT

Individual read raw sequences have been submitted at the Sequence Read Archive (SRA) (Project Accession Number: SRP149671).
