# Supplementary figures and images for: Impact of supplementation on deleterious mutation distribution in an exploited salmonid
Source: Evol Appl. 2018 Jul 1;11(7):1053–65. doi: 10.1111/eva.12660 (PMC6050184; doi:10.1111/eva.12660)

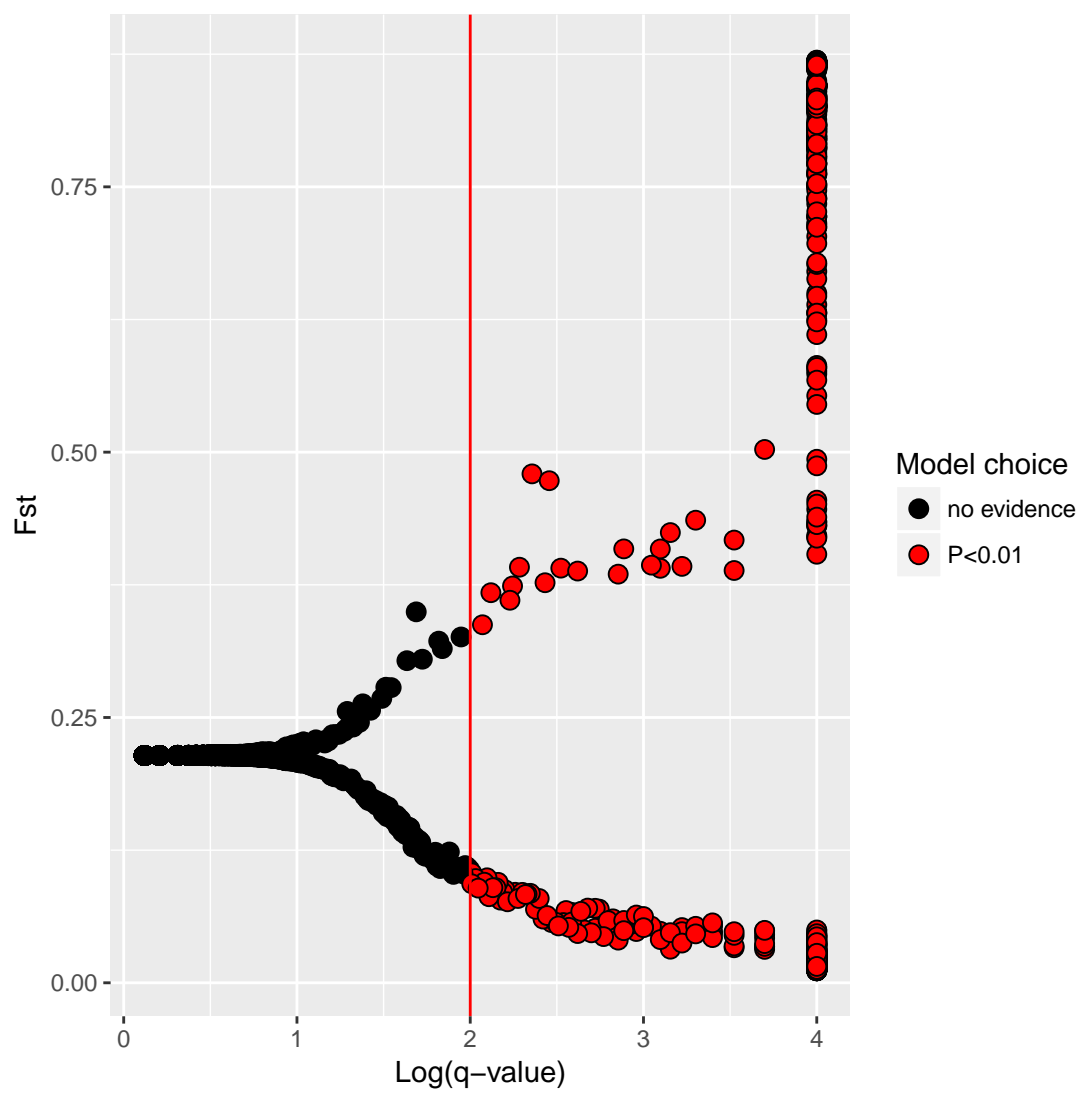

Supplement: Supplementary file 1 [file EVA-11-1053-s001.pdf]
