# Supplementary material for: Impact of supplementation on deleterious mutation distribution in an exploited salmonid
Source: Evol Appl. 2018 Jul 1;11(7):1053–65. doi: 10.1111/eva.12660 (PMC6050184; doi:10.1111/eva.12660)

**Detection of putative outliers.**

1. Bayescan

BAYESCAN estimates population-specific *F*_ST_ coefficients by the Bayesian method described in Beaumont & Balding (2004) and uses a cut-off based on the mode of the posterior distribution to detect SNPs under selection (Foll & Gaggiotti 2008). SNPs with a posterior probability over 0.95 were considered as outliers, after running 100,000 iterations. We specified a ‘prior’ odd of 10,000, which sets the neutral model being 10,000 times more likely than the model with selection to minimize false positives as suggested by Lotterhos & Whitlock (2014).

**Figure S1.1:** *F*_ST_ BAYESCAN estimates. Out of 4,982 SNPs, 437 were detected as being under divergent selection (red points with highest *F*_ST_ estimates) and 722 were detected as being under balancing selection (red points with lowest *F*_ST_ estimates)

1. Random forest

Secondly, because different temperature variables may impose different selective pressures on Lake Trout (Perrier et al. 2017), we produced a principal component analysis (PCA) on maximum, maximum winter, mean, minimum summer and minimum annual temperature of each site. Based on a broken-stick distribution, only the first axis was considered meaningful (91.4% of the variation; loading factors: maximum = 0.32; maximum winter = 0.47; mean = 0.44; minimum summer = 0.41; minimum = 0.55) and kept for further analyses (Legendre and Legendre, 1998). The random forest algorithm was implemented to identify SNPs that were associated with the first temperature PC using the function ‘*randomForest*’ of the R package ‘randomForest’ v4.6-12. Since population structure can bias the accuracy of the association, the genotypes were adjusted (Zhao et al. 2012; Brieuc et al. 2015) as follows: all genotypes were converted to 0 (aa), 1 (ab) or 2 (bb) and a generalized linear model was fitted against the admixture proportions quantified with the software ADMIXTURE v 1.3.0 (Alexander et al 2009). ADMIXTURE was performed over the entire SNP dataset from a K ranging from 2 to 30. Since two best K (21 and 24) were detected by ADMIXTURE, we ran two independent analyses with the genotype residuals obtained with the admixture proportions corrections under both K values. In each case, 100,000 trees were computed using all SNPs to determine the “importance” of each locus. The “importance” of a SNP is an indicator of how a SNP in interaction with other SNPs is associated with the first temperature PC. To select a set of ‘important SNPs’ for a given analysis, we examined the distribution of importance of SNPs and used the upper end of the elbow as the cut-off (Goldstein et al. 2011; Laporte et al. 2015; Laporte et al. 2016). Finally, we used the function *‘rfcv*’, from the same R package, to produce a cross validation based on a backward purging approach on the selected important SNPs (Holliday et al. 2012). For further analyses, only important SNPs retained in both analyses were used. Consequently, we removed SNPs detected as putatively under divergent and balancing selection and/or polygenic selection related to temperature from the entire SNPs dataset and remaining SNPs were considered as “neutral SNPs.

List of SNPs identified as putatively under polygenic selection related to temperature for K= 21 and K = 24 random forest analysis


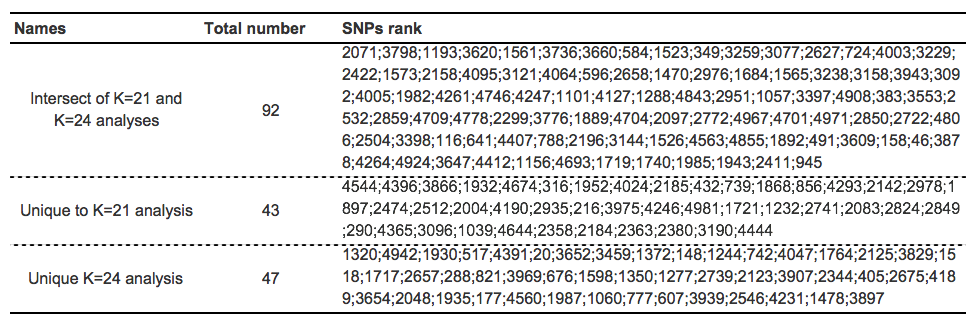

Supplement: Supplementary file 3 [file EVA-11-1053-s003.docx]
