# Supplementary material for: Impact of supplementation on deleterious mutation distribution in an exploited salmonid
Source: Evol Appl. 2018 Jul 1;11(7):1053–65. doi: 10.1111/eva.12660 (PMC6050184; doi:10.1111/eva.12660)
Supplement: Supplementary file 4 [file EVA-11-1053-s004.docx]

***Comparison SNPs/microsatellites***

SNPs database produced in our main manuscript contained the exact same individuals that the one of Valiquette et al. (2014) based on 19 microsatellites. We thus took advantage of both of the large dataset to compare their respective capacity to detect an effect of stocking on neutral genetic variation. This supplementary material aims to present all the details for Material and Methods as well as Results and Discussion about this markers comparison.

***2.Material & Methods***

*2.1 Removing markers under potential selection*

Genetic diversity, differentiation and structure were assessed and compared between SNPs and microsatellites based on putative neutral markers. The detection of putative outliers was performed with BAYESCAN v.2.1 (Foll & Gaggiotti 2008) (see manuscript for SNPs) The putative outliers were subsequently removed from datasets, such that only putative neutrals remained (hereafter called “neutral markers”) for the subsequent analyses. Details of Bayescan are provided in Supplementary Material 1.

*2.2 Diversity*

For neutral microsatellites, allelic richness was calculated using the R-package ‘PopGenReport’ (Adamack and Gruber 2014) with the rarefication correction for sample size (El Mousadik & Petit, 1996). GENEPOP version 4.2 (Raymond & Rousset 1995; Rousset 2008) was used to estimate observed and expected heterozygosities (H_O_ and H_E_ respectively) and a *t*-test was performed between H_E_ of unstocked *versus* stocked populations. Relationships between diversity estimates from the two neutral marker types were tested with a linear regression performed between (i) H_E_ from neutral microsatellites and SNPs and (ii) microsatellites mean allelic richness and number of polymorphic neutral SNPs.

*2.3 Neutral genetic differentiation and structure*

The R-package assigner v.0.3.9 (Gosselin et al 2016) was used to estimate pairwise *F*_ST_ (Weir &Cockerham 1984) across all populations and tested for significance with 1,000 bootstraps. *F*_ST_ estimations were thus performed on the datasets. A Mantel test was performed to calculate pairwise correlation among the two *F*_ST_ matrices. Estimations of neutral admixture proportions were performed with a discriminant analysis of principal components (DAPC). Then, the function ‘*find.clusters*’ from the ‘adegenet’ R package version 2.0.1 (Jombart & Ahmed 2011) was used to assess the optimal number of groups with the Bayesian information criterion (BIC) method, considering no *prior* on group individual populations and allowing a maximum of 30 clusters. Finally, the posterior individual assignment probabilities to each group (Q matrix) was obtained running the ‘*dapc*’ function using the optimal number of discriminant functions to retain the optimal α–score obtained from each dataset (Jombart et al. 2010).

*2.4 Detecting the effect of stocking on patterns of neutral genetic structure*

As for SNPs neutral (see manuscript), db-RDAs were produced on neutral microsatellites pairwise *F*_ST_ distance matrix. In addition, PCA were produced on Q matrix to serve as proxies for genetic structure. In this way, we tested if the admixture proportions (Q matrix of a DAPC averaged at population level) of the microsatellites and neutral SNPs could be impacted by previously explained PC-axes related to stocking and spatial autocorrelation (db-MEMs) with RDAs. The ‘*ordistep*’ function was used to select the model including the best explanatory variables among db-MEMs and stocking PC-axes and partial RDAs were produced to estimate PVE explained by PCs stocking after controlling for spatial autocorrelation.

***3. Results***

*3.1 Diversity*

One microsatellite under potential divergent selection was also removed (see manuscript for SNPs). The median estimates of the observed and expected heterozygosity (H_O_ and H_E_) were respectively 0.20 and 0.18 for the neutral SNPs and 0.77 and 0.78 for microsatellites (Table 2). The mean allelic richness for microsatellites (Ar) ranged from 2.2 (pop HA) to 4.5 (pop PM), with a median value of 4 (Table 2). Highly significant positive correlations were observed between (i) H_E_ from microsatellites and neutral SNPs (adj. R^2^ = 0.76; P< 0.001) and (ii) between mean microsatellite allelic richness (Ar) and the number of neutral polymorphic SNPs (Adj. R^2^ = 0.59; P < 0.001). The mean H_E_ for both microsatellites and neutral SNPs were significantly higher among stocked populations (mean H_E microsatellites_ = 0.82, mean H_ESNPs_ = 0.19) than among unstocked populations (mean H_E microsatellites_ = 0.71, mean H_ESNPs_ = 0.16; P _t.test for microsatellites_ = 0.031 and P _t.test for SNPs_ = 0.016).

*3.2 Genetic differentiation and structure*

A pronounced pattern of population structure was generally observed among populations, both for microsatellite and SNP datasets. For microsatellites, pairwise population differentiation (*F*_ST_) ranged from 0.08 to 0.40 (mean = 0.11) whereas mean pairwise *F*_ST_ per population (that is the average of pairwise comparisons with all other populations) ranged from 0.07 (pop PM) to 0.29 (pop HA). For neutral SNPs, pairwise *F*_ST_ ranged from 0.01 to 0.50 (mean = 0.22) and mean pairwise *F*_ST_ per population ranged from 0.15 (pop GR) to 0.36 (pop HA). Distributions of pairwise *F*_ST_ for each SNP category are presented in Figure S2.1. Furthermore, Mantel test on *F*_ST_ estimations showed a significant correlation between microsatellites and neutral SNPs (r = 0.82; CI95[0.49-1.00]; P < 0.001).

***Figure S2.1:*** *Boxplots of pairwise F_ST_ across the Lake Trout populations estimated for microsatellites, neutral SNPs, and putative deleterious SNPs. Populations are ordered by increasing stocking intensity within administrative regions (from West to East).*

The DAPC confirmed the pronounced genetic structure among populations. For both sets of markers (microsatellites and neutral SNPs) the BIC values decreased as the number of K increased until K = 24 (Figure S2.2).

***Figure S2.2:*** *Values of Bayesian Information Criteria (BIC) along the number of clusters inferred in the DAPC (from K = 2 to K =30), for microsatellites, neutral and all SNPs.*

Thus K=24, which also corresponds to the total number of sampled populations, was used to estimate the posterior individual probabilities of assignment (Q matrix; Figure S2.3). On average, microsatellites provided a lower percentage of correctly assigned individuals to their population of origin than the SNPs dataset, with a median value of the average individual admixture proportion of 56% (mean = 62%), ranging from 29% (TZ lake) to 100% (HA lake). The same average estimate for neutral SNPs has a median of 99 % (mean = 93%), ranging from 53% (ES lake) to 100 % (AC, BO, CA, GR, KI, MA, MG, OF, PB, TU, WA lakes).

***Figure S2.3:*** *DAPC barplots results for microsatellites, neutral SNPs and all SNPs. Each bar corresponds to individual proportion of assignment to one or different sources of stocking. Populations are ordered by increasing stocking within administrative regions (from West to East).*

*3.3 Detecting the effect of stocking on patterns of neutral genetic structure*

For pairwise *F*_ST_ distance matrix, five PCo-axes explaining at least 5% of the variance were retained for microsatellites and neutral SNPs. These PCo-axes explained a total of 46% and 60% of total variance respectively. For Q matrix, seven PC-axes (microsatellites; total of 64% of variance) and 12 PC-axes (neutral SNPs; total of 61% of variance) explaining at least 5% of the variance were kept. Variables selected by backward selection, PVE of the global model, and PVE of stocking after controlling for spatial correlation are presented in Table S2.1. Maximum variance inflation factors were all under 6 (with majority under 2), which is also under the suggested threshold of 10 (Hair et al. 1998). Together, the results show a slight increase of PVE explained by stocking in the partial model when population average Q matrix and pairwise *F*_ST_ distance were calculated with neutral SNPs relative to estimations from microsatellites (Table S2.1). Accordingly, the number of stocking PC-axes selected in the global model also increases when population average Q matrix were estimated with neutral SNPs compared to microsatellites (Table S2.1).

**Table S2.1:** Results of redundancy analyses on admixture proportion genetic differentiation testing for the effect of stocking and spatial auto-correlation on microsatellites and neutral SNPs. The four stocking variables are PC-axis surrogating stocking intensity (PC-1 was correlated to the lakes sources 31-miles and Blue Sea; PC-2 to lake Tremblant; PC-3 to Mitis lake; PC-4 no correlation > 0.6). Spatial autocorrelation was estimated with distance-based Moran’s eigenvector Map (db-MEM), which correspond to one of the 16 axes obtained from latitude, longitude and altitude coordinates. Percentage of variation explained (PVE) corresponds to the adjusted R_2_ and was obtained from all variables in the model (stocking and db-MEMs) and from partial analyses for stocking (see manuscript for more details).

| **Genetic differentiation (pairwise *F*_ST_)** | **Stocking** | **db-MEMs** | **PVE** | **PVE Stocking** | |
| --- | --- | --- | --- | --- | --- |
| 18 Microsatellites | PC-3 | 3,6,9,14 | 27.0** | 13.7** | |
| 3557 neutral SNPs | PC-3 | 2,9,14 | 24.5*** | 15.7** | |
| **Admixture proportion  (population average Q matrix)** |  |  |  |  | |
| 18 Microsatellites | PC-1,2,3 | 2,6,10,14 | 30.3*** | 13.2** |  |
| 3557 neutral SNPs | PC-1,2,3,4 | 1,2,10,12,14 | 26.5*** | 16.9*** |  |

P-value = ^#^<0.1; **: <0.01; *** :<0.001.

**4. Discussion**

Several studies have previously conducted comparisons between microsatellites and SNPs (Liu et al. 2005; Vali et al. 2008; Coates et al 2009; DeFaveri et al. 2013; Rasic et al 2014; Moore et al. 2014; Bradbury et al. 2015; Elbers et al. 2016; Vendrami et al 2017). However, the present study is one of the very few to control for sampling (*i.e.* genotyping the exact same individuals) before comparing the performance of both markers based on a large sampling (578 individuals from 24 localities) and large putative neutral SNPs (3557) and microsatellites (18) datasets. Our study shows that expected heterozygosity estimated using microsatellites and SNPs were highly correlated although higher for microsatellites as expected based on the know high mutation rate of microsatellite loci (Vignal et al. 2002). Pairwise *F*_ST_ values were larger for SNP markers than for microsatellites, which is also expected given the intrinsic mathematical dependence of *F*_ST_ on heterozygosity, number of alleles and their frequency (Hedrick 2005; Jakobsson et al 2013). Nevertheless, a strong correlation between *F*_ST_ estimates from microsatellites and SNPs was observed, indicating that genetic differentiation estimated by both markers represents similar historical (*e.g.* colonization) and contemporary (*e.g.* drift, migration by supplementation) processes. Finally, SNPs demonstrated higher power in resolving Lake Trout genetic structure, confirming the previously mentioned advantage of SNPs over microsatellites to define population structure (Liu et al. 2005; Moore et al. 2014; Bradbury et al. 2015). More specifically, DAPC with neutral SNPs revealed a clear separation of distinct genetic clusters, while the 18 polymorphic microsatellites showed much less resolution in delineating populations, along with a lower individual proportion of assignment to the population of origin. Nonetheless, despite SNPs better define population structure, no major discrepancy was observed between both datasets. Finally, SNPs find a slight increase in the effect of stocking on both pairwise genetic differentiation and Q matrix, as well as the detection of the effect of one more stocking variable for the Q matrix. Overall, it suggests that SNPs is slightly more sensible to detect the impact of stocking in the wild.
